# Roads to advanced vaccines: influenza case study

**DOI:** 10.1111/1751-7915.12835

**Published:** 2017-08-15

**Authors:** Peggy Riese, Carlos A. Guzmán

**Affiliations:** ^1^ Department of Vaccinology and Applied Microbiology Helmholtz Centre for Infection Research D‐38124 Braunschweig Germany

## Abstract

Vaccines represent a cornerstone to ensure healthy lives and promote well‐being for all at all ages. However, there are many diseases for which vaccines are not available, are relatively ineffective or need to be adapted periodically. Advances in microbial biotechnology will contribute to overcoming these roadblocks by laying the groundwork for improving and creating new approaches for developing better vaccines, as illustrated here in the case of influenza.

The 3rd Sustainable Development Goal – Ensure healthy lives and promote well‐being for all at all ages – includes the target of developing vaccines for infectious diseases. Influenza A and B viruses can cause acute respiratory tract infections and result in annual epidemics associated with high economic burden, increased hospitalization and up to 500 000 deaths worldwide (WHO, 2016). Estimates from a study in the United States placed annual direct medical costs for influenza epidemics at an average of $10.5 billion, lost earnings at an average of $16.3 billion, and total economic burden at an average of $87.1 billion (Molinari *et al*., 2007). Vaccination represents the most cost‐efficient tool to prevent and control influenza infections by reducing viral transmission and limiting the severity of infection. The currently licensed seasonal influenza vaccines which encompass live attenuated, inactivated and recombinant vaccine approaches (reviewed by Houser *et al*.) are mainly focused on the generation of neutralizing antibodies specific for the viral hemagglutinin (HA) and neuraminidase (NA) surface proteins (Houser and Subbarao, 2015). These vaccines are usually most effective in healthy young adults but with only about 60% protection. Thus, several challenges need to be tackled until the optimal influenza vaccine is designed. The major bottleneck of the current influenza vaccines is their lack of cross‐reactivity against non‐matched influenza strains. Antigenic drift and shift of the circulating influenza strains require annual evaluation and adaptation of seasonal vaccines rendering their manufacturing time‐consuming and expensive. The selection of strain‐specific antigen or virus variants to be implemented in the seasonal vaccine formulation is based on global surveillance and predictions that need to be made several months ahead allowing all necessary manufacturing steps including amplification, inactivation, purification and bottling (WHO, 2016). The H1N1 pandemic in 2009 strongly demonstrated the arising problems to produce a vaccine against a new emerging influenza virus in a short time period as a second outbreak could not be prevented (Lee *et al*., 2014). One approach to handle these issues would be to stockpile vaccines generated against different virus variants and subtypes with pandemic potential. However, this requires a precise selection of promising candidates and their subsequent testing in preclinical and clinical trials (Yen *et al*., 2015).

The difficulties in predicting the influenza strains causing the next seasonal or pandemic outbreak increase the demand for a ‘universal’ vaccine aimed at inducing broadly cross‐reactive immunity independent of antigen shift or drift and thereby rendering annual vaccination unnecessary (Krammer and Palese, 2014). In the last decade, various approaches were investigated and some promising vaccine candidates have already entered into the clinical phases (Soema *et al*., 2015). One overall approach is focused on enhancing antibody responses as well as the generation of more broadly reactive antibodies. Instead of developing vaccines which target the highly immunogenic, but also very variable influenza surface proteins HA or NA, the focus shifts towards the more conserved viral components. In this context, the highly conserved stalk region of the HA protein was discovered as a potential vaccine candidate (Gomez Lorenzo and Fenton, 2013). In contrast to the head‐HA antibodies, stalk antibodies cannot bind to the receptor and therefore not block receptor binding of the virus. However, these antibodies inhibit viral membrane fusion and maturation and can mediate antibody dependent cytotoxicity. Antibodies specific for the conserved HA stalk region were reported to induce broad neutralization and target multiple influenza strains (Krammer and Palese, 2013; Margine *et al*., 2013). Although antistalk‐antibodies can be observed after natural influenza infection, their detection following vaccination is difficult, which renders the evaluation of vaccine success complicated. Moreover, in‐depth safety studies are required as recent findings reported that the development of respiratory disease symptoms in pigs due to HA2 stalk‐reactive antibodies mediated viral fusion following vaccination (Khurana *et al*., 2013). Another approach is focused on targeting the matrix protein 2 (M2), a transmembrane protein which acts as an ion channel and is crucial for the release of viral material into the host cells. The M2 sequence is highly conserved between the different influenza subtypes, and immune responses against M2 are suggested to display high cross‐reactivity. Similar to the HA stalk approach, M2‐targeting vaccines are not able to prevent infection itself but inhibit viral replication and therefore spreading within the host. A completely different approach makes use of computational tools. Here, the vaccine consists of a computationally optimized broadly reactive HA antigen (COBRA) expressed by a virus‐like particle (VLP). The COBRA HA sequence consists of a consensus sequence of a variety of influenza viruses harbouring the most common amino acid at each position. A COBRA HA‐containing vaccine was demonstrated to induce broadly reactive antibodies against multiple clades of H5N1 viruses and to induce less pathology in non‐human primates as compared to a non‐consensus vaccine (Giles and Ross, 2011; Giles *et al*., 2012). The utilization of viral vectors which (i) express high levels of influenza antigens, (ii) target specific cell types and (iii) can be delivered by different routes is a promising alternative method. One major advantage of vector‐based vaccines is their capacity to induce cross‐reactive cellular immunity. Cytotoxic T cells mainly recognize highly conserved, internal antigens. Although they are not able to prevent an infection, they directly kill virus‐infected cells and activate further crucial antiviral innate and adaptive immune players via cytokine release, thereby contributing to diminish disease severity and the duration of infection (Houser and Subbarao, 2015; de Vries and Rimmelzwaan, 2016). Thus, efforts aiming at designing cross‐protective influenza vaccines can no longer neglect the potential of cellular immunity. Current preclinical and clinical studies are focused on modified vaccinia Ankara and adenoviral vectors expressing the highly conserved internal nucleoprotein and the surface matrix 2 proteins which were shown to induce cross‐reactive T‐cell responses (Gurwith *et al*., 2013; Antrobus *et al*., 2014; Kreijtz *et al*., 2014). However, the potential induction of tolerance by antivector immunity needs to be kept in mind. The implementation of adjuvants and the application of different vaccination strategies represent additional tools to enhance and broaden vaccine‐induced immunity. Adjuvants added to a vaccine formulation enable to (i) increase the immunogenicity, (ii) reduce the amount of antigen and number of doses needed and (iii) fine‐tune immune responses in a favoured direction. Their immune‐stimulatory effects are mainly mediated by physical or chemical stabilization of the antigen as well as enhanced antigen delivery, processing and presentation (Riese *et al*., 2013). So far, three different adjuvants were incorporated in seasonal or pandemic influenza vaccines, aluminium hydroxide, MF59^®^ and AS03, and all were proven to be superior to the non‐adjuvanted versions (Tetsutani and Ishii, 2012). However, more research needs to be focused on their mechanism of action, pharmacokinetics and dynamics and their safety profile. The identification of adjuvants acting by the mucosal route might represent a cornerstone for the development of influenza vaccines which can be administered intranasally and prevent infection at the site of pathogen entry (Soema *et al*., 2015; Schulze *et al*., 2016). Additionally, several studies reported the superiority of prime/boost schedules with regard to vaccine efficacy (Van Reeth *et al*., 2017). However, the public acceptance of prime/boost strategies especially for seasonal vaccination needs to be verified.

Despite the ongoing efforts and progress in developing a ‘universal’ vaccine, many hurdles still need to be overcome, and it is doubtful that this aim will be achieved in the next decades. The design of influenza vaccines stratified as personal medicine needs to be taken into consideration, especially for those populations which are highly vulnerable to influenza infection with severe disease outcome like young children, pregnant women, elderly or immunocompromised patients (WHO, 2016). The elderly represent a continuously growing part of the population displaying specific vaccine requirements. Of the total burden cost, 64% is attributable to the elderly (≥ 65 years) (Molinari *et al*., 2007). As current influenza vaccines are tailored rather for adults aged between 18 and 49 years, these vaccines are not perfectly adjusted to the elderly as they neglect the high occurrence of underlying chronic diseases and decreased immunity with increasing age, also referred to as immunosenescence (Lang *et al*., 2012). In contrast to healthy young adults, the efficacy of standard influenza vaccines is very poor in elderly. Existing approaches to enhance the vaccine‐induced immunogenicity for elderly are focused on either raising the antigen dose or adding adjuvants to the vaccine formulation (Song *et al*., 2013; Tsai, 2013; DiazGranados *et al*., 2014; Shay *et al*., 2017). These are already on the market. For example, the inactivated influenza vaccine Fluad, approved in Europe for individuals ≥ 65 years, is adjuvanted with the oil in water emulsion MF59 and was proven to enhance immunogenicity (Domnich *et al*., 2017). However, a more in‐depth understanding of immunosenescence as well as the impact of underlying chronic diseases on vaccination outcome is urgently required. Thus, the comparison of immunological and molecular signatures between young and elderly might provide valuable ideas to fine‐tune already existing or design more powerful new vaccines (Kennedy *et al*., 2016; Sobolev *et al*., 2016; Castrucci, 2017). The implementation of systems biology approaches, which combine system‐wide measurements and predictive modelling, will beneficially support vaccine development for individual groups and further help to define predictive biomarkers for vaccine responsiveness (Nakaya *et al*., 2011, 2015; Hagan *et al*., 2015).

The determination of an accurate correlate of protection is a prerequisite to reliably define the efficacy of influenza vaccines or potential candidates. Currently, a HAI titre of ≥ 1:40 correlates with 50% protection from infection in healthy adults. However, a higher titre might be required in the high‐risk groups (Black *et al*., 2011; Haq and McElhaney, 2014). Further, seasonal and pandemic live attenuated influenza vaccines induce protection in the absence of humoral responses rendering the HAI titre not the most meaningful measurement (Sridhar *et al*., 2013; Coelingh *et al*., 2014). A more global correlate of protection which also considers virus‐specific cellular immune parameters is needed to evaluate the protective potential of influenza vaccines. In this line, several clinical studies demonstrated a correlation of elevated virus‐specific CD4 and CD8 T‐cell responses and decreased viral shedding and influenza‐related illness (Wilkinson *et al*., 2012; Altenburg *et al*., 2015; Wang *et al*., 2015).

The development of novel, more advanced influenza vaccines requires not only immunological or molecular knowledge but also improved biotechnological skills like the generation of optimized synthetic antigens or improved formulations, as well as the establishment of novel production strategies. In fact, the production of inactivated and live attenuated influenza vaccines still mainly relies on the use of embryonic eggs (Houser and Subbarao, 2015). Beside their allergic potential and ethical concerns, their availability/access in case of a sudden pandemic outbreak is limited. Therefore, revised approaches are aiming at egg‐independent manufacturing. Thus, the usage of mammalian or insect cells is increasingly coming into focus and has been proven to be sufficient for the production of licensed inactivated (Flucelvax^®^ and Celvapan^®^) or recombinant (FluBlok^®^) vaccines, respectively, thereby offering alternative egg‐free approaches (Milian and Kamen, 2015). Furthermore, efforts to introduce DNA‐ or RNA‐based vaccines, aimed at replacing whole cell vaccine approaches in the future, will also contribute to the development of egg‐free vaccine manufacturing processes. To create vaccine stockpiles, the shelf life and stability of seasonal influenza vaccines, which are so far restricted to approximately one year, need to be enhanced. In addition, the need of a cold‐chain during distribution and storage is expensive and difficult to guarantee especially in developing countries (Soema *et al*., 2015). Therefore, development of tools aiming at improving antigen stability and driving freeze‐drying manufacturing processes by including excipients to the formulation would greatly improve the shelf life, transport and storage duration of influenza vaccines (Geeraedts *et al*., 2010; Soema *et al*., 2014; White *et al*., 2016).

Altogether, the design of advanced influenza vaccines protecting all age and vulnerable groups is a challenging and ongoing process. Several promising candidates are on the road to success. However, research needs to be continuously focused on (i) understanding immune responses to vaccines in the different populations, (ii) improving vaccine formulations and manufacturing and (iii) defining predictive markers for vaccine efficacy.

Next to influenza, a number of other infectious diseases require intense vaccine developmental research. In particular, the third world and threshold countries are confronted with diseases like HIV, malaria and tuberculosis, which are the leading causes of death. Solutions need to be defined by public and private sectors that enable providing the most‐efficient access to vaccines as well as supporting vaccine development primarily in the affected countries. Further, joint efforts are required to advance the global vaccine coverage, especially for vaccines against Haemophilus influenza type B, rubella, pneumococcus and rotavirus recommended for children. Here, the vaccine coverage is below 50–15% respectively (Rappuoli *et al*., 2015). Improving vaccines and their coverage is considered to strongly influence the 3rd Sustainable Development Goal – Ensure healthy lives and promote well‐being for all at all ages. A major cause of poverty is related to infectious diseases. Disease prevention will improve life quality and thereby enhance the contribution to the community by each individual. For example, children which are not confronted with infections and the subsequently sequelae can achieve a better education. Furthermore, the time and costs of care taking is reduced and opens new opportunities for adults in terms of education and work contribution. Summarized, improving vaccines will substantially contribute to social and economic growth; good health makes people satisfied and productive.

## Conflict of Interest

None declared.
